# Krüppel-like Factor 2 (KLF2) Regulates Autophagy, Mitophagy, Mitochondrial Health, and Function During Foam Cell Formation

**DOI:** 10.54457/dr.202601003

**Published:** 2026-03-13

**Authors:** Md Sariful Islam Howlader, Manjusri Das, Surajit Hansda, Prathyusha Naidu, Hiranmoy Das

**Affiliations:** 1 Department of Pharmaceutical Sciences, Jerry H. Hodge School of Pharmacy, Texas Tech University Health Sciences Center, Amarillo, Texas 79106, USA

**Keywords:** Foam cell, Kruppel-like factor 2, Autophagy, Mitophagy, Glycolysis

## Abstract

**Backgrounds:**

Foam cell (FC) formation is a hallmark of early atherosclerosis, driven by dysregulated lipid uptake, impaired mitochondrial clearance, and metabolic reprogramming in myeloid cells. However, the precise role of KLF2 in modulating autophagy, mitophagy, and glycolysis during FC formation remains inadequately explored.

**Methods:**

This study uncovers the critical regulatory role of Krüppel-like factor 2 (KLF2) during foam cell formation of myeloid cells (RAW264.7) using quantitative real-time PCR, immunocytochemistry, confocal microscopy, and glycolysis stress test methods.

**Results:**

Exposure to oxidized low-density lipoprotein (ox-LDL) suppressed autophagy and mitophagy markers in myeloid cells. It also increased glycolytic activity in myeloid cells during FC formation. A well-known chemical suppressor of KLF2, GGPP, further amplified these changes, highlighting the importance of endogenous KLF2 in maintaining mitochondrial health and metabolic functions during formation. To confirm the role of KLF2 in this process, when a chemical inducer of KLF2, GGTI298, was added during FC formation, it restored autophagic and mitophagic machinery, including the expression of Beclin1, LC3B, Parkin, and Pink1, and reversed the abnormal increase in glycolysis during FC formation.

**Conclusion:**

These findings demonstrate that KLF2 is a key transcriptional regulator that limits FC formation by preserving mitochondrial health and reducing excessive glycolysis. Furthermore, these results suggest KLF2 could be a target for future development of therapeutics for preventing FC formation, which is an early event in atherosclerosis development.

## Graphical Abstract

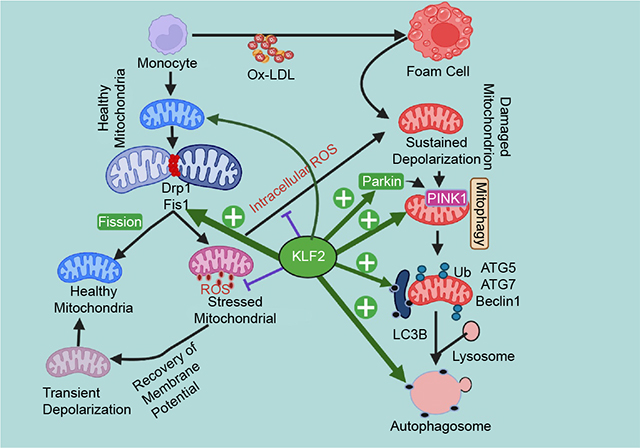

## Introduction

Atherosclerosis remains a leading cause of morbidity and mortality worldwide, primarily through complications such as myocardial infarction and stroke. However, atherosclerotic pathogenesis is rooted in chronic inflammation and lipid accumulation within the intima of large and medium-sized arteries, leading to the formation of atherosclerotic plaques. A hallmark of early lesion development is the formation of macrophage-derived foam cells (FC), which emerge from the uncontrolled uptake of oxidized low-density lipoprotein (ox-LDL) and a failure to effectively manage intracellular lipid overload and cellular debris^[1,2]^.

Autophagy is an evolutionarily conserved cellular process that plays a pivotal role in maintaining intracellular homeostasis by degrading and recycling damaged organelles, misfolded proteins, and excess lipids through the lysosomal pathway. This catabolic mechanism is essential for cellular survival under conditions of metabolic stress, nutrient deprivation, and oxidative injury^[3]^. Mitophagy, a selective form of autophagy targeting dysfunctional mitochondria, is particularly important for preserving mitochondrial quality control and reducing mitochondrial-derived reactive oxygen species (ROS), which can otherwise exacerbate inflammation and cellular injury^[4,5]^. In the context of foam cell (FC) formation, a marked suppression of autophagic and mitophagic activities has been observed, contributing to lipid accumulation, impaired mitochondrial dynamics, and the persistence of damaged organelles, which collectively promote a pro-atherogenic environment^[6,7]^. Furthermore, defective autophagy impairs cholesterol efflux and enhances inflammasome activation, thereby driving plaque progression and instability^[8]^. The importance of these clearance pathways is further underscored by findings showing that pharmacological or genetic restoration of autophagy/mitophagy in monocytes not only reduces lipid burden but also suppresses inflammatory responses, preserves mitochondrial function, and stabilizes atherosclerotic plaques^[9,10]^. Therefore, elucidating the regulatory networks that govern autophagy and mitophagy during FC development is crucial for identifying novel therapeutic targets aimed at modulating metabolic stress and immune dysfunction in the context of atherosclerosis.

FC formation involves a significant metabolic reprogramming, where myeloid cells in atherosclerotic environments show increased glycolytic activity, similar to the metabolic profile of classically activated M1 macrophages^[11]^. This shift promotes proinflammatory gene expression while reducing mitochondrial respiration, collectively fostering a pro-atherogenic phenotype. Additionally, autophagy and mitophagy, essential processes for lipid breakdown and maintaining mitochondrial health, are decreased in foam cells, leading to lipid buildup through efficient uptake, ROS production, and the development of ineffective cellular clearance mechanisms, which drive plaque progression^[6,12]^.

Krüppel-like factor 2 (KLF2), a zinc-finger transcription factor, plays a crucial role in maintaining cellular homeostasis, especially in endothelial and immune cells. Initially, KLF2’s role was recognized for its vasoprotective effects in endothelial cells, mediating anti-inflammatory, anti-thrombotic, and anti-oxidative signaling. KLF2 expression is also tightly regulated in myeloid cells during inflammation^[13,14]^. Notably, KLF2 levels are downregulated during myeloid cell differentiation, and its loss has been linked to enhanced ox-LDL uptake, leading to increased FC formation^[15]^. Conversely, statins and other agents have been found to reduce the atherosclerotic burden, partly through the restoration of autophagic flux and endothelial integrity^[16,17]^. Unlike the statin-KLF2-autophagy relationship described in endothelial cells, our study demonstrates this regulation for the first time in monocyte-derived foam cells. Emerging reports suggest that KLF2 serves as a crucial molecular link between metabolic regulation and intracellular clearance mechanisms in various cell types. For example, KLF2 has been shown to induce the expression of Beclin1 and LC3 in vascular smooth muscle cells, facilitating autophagy under various oxidative stress conditions^[14,18–25]^. However, the precise role of KLF2 in modulating glycolysis, autophagy, and mitophagy during FC formation remains inadequately explored.

In this study, we hypothesize that KLF2 may act as a master regulator of myeloid cell metabolism and mitochondrial homeostasis during FC formation. Previous studies have established that pharmacological modulation of the isoprenoid metabolic pathway strongly influences KLF2 expression. For example, statins induce KLF2 and exert vasculoprotective effects^[26,27]^. In contrast, geranylgeranyl pyrophosphate (GGPP) suppresses KLF2 expression, whereas geranylgeranyl transferase inhibitors, such as GGTI-298, enhance KLF2 expression by inhibiting protein prenylation^[27]^. Beyond vascular contexts, KLF2 has been identified as a critical transcription factor regulating endothelial inflammatory activation^[13]^, monocyte proinflammatory responses^[14]^, and autophagy-mediated osteoclastogenesis^[23]^. These studies provide a solid foundation for investigating how modulation of KLF2 with GGPP and GGTI-298 impacts glycolysis, autophagy, and mitophagy during ox-LDL–induced FC formation.

## Materials and Methods

### Reagents and antibodies

The RAW264.7 murine myeloid cell line was obtained from the American Type Culture Collection (ATCC, #TIB-71). Antibodies specific to DRP1 (#ab184247), and ATG5 (#ab12994) were purchased from Abcam. Additional autophagy and mitophagy-related antibodies, including Beclin1 (#4122S), ATG7 (#8558S), LC3B (#12741S), PINK1 (#6946S), and PARKIN (#2132S), were obtained from Cell Signaling Technology. The FIS1 antibody (#10956–1-AP) was sourced from Proteintech. Biochemical reagents used in the study included the BCA Protein Assay Kit (#23225) and Oxidized Low-Density Lipoprotein (ox-LDL, #L34357), both from Thermo Fisher Scientific. Paraformaldehyde (4%, #sc-281692) was purchased from Santa Cruz Biotechnology, while Triton X-100 (#T8787), RIPA lysis buffer (#20–188), and bovine serum albumin (BSA, 5%, #A4737) were acquired from Sigma-Aldrich. Additional molecular biology reagents included TRIzol reagent (#15596026) and DAPI nuclear stain (#D1306) from Invitrogen, and the High-Capacity RNA-to-cDNA Kit (#4387406) and SYBR Green PCR Kit (#4309155) from Applied Biosystems.

### Cell culture and FC induction

The murine macrophage cell line RAW264.7 was obtained from the American Type Culture Collection (Cat # TIB-71, ATCC) and cultured in Dulbecco’s Modified Eagle Medium (DMEM; Gibco, Cat # 11–965-092) supplemented with 10% fetal bovine serum (FBS; Cat # A3840001, Thermo Fisher Scientific, HyClone), 1% penicillin-streptomycin, Cat # 15140122, Invitrogen), and maintained at 37 °C in a humidified atmosphere of 5% CO_2_. For FC induction, cells were seeded at a density of 1 × 10^6^ cells/well in 6-well plates and incubated with oxidized low-density lipoprotein (ox-LDL, 50 μg/mL; Cat # L34358 Thermo Fisher Scientific) for six days unless otherwise specified.

### Chemical modulation during FC formation

To examine the effects of pharmacological modulation during FC formation, chemical agents targeting the geranylgeranylation pathway were employed. For suppression of geranylgeranylation-dependent signaling, cells were treated with GGPP (10 μM; CAS No. 313263–08-0; Cayman Chemical). GGPP was added 1 hour before ox-LDL exposure and maintained throughout the experimental period. Foam cell formation was induced by treatment with oxidized low-density lipoprotein (ox-LDL; 50 μg/mL), which was maintained for six days.

For inhibition of protein geranylgeranyltransferase activity, cells were treated with GGTI298 (20 μM; CAS No. 1217457–86-7; Cayman Chemical) during ox-LDL–induced foam cell formation. All treatments were maintained for the duration of the experiment unless otherwise specified. This experimental design allowed assessment of transcriptional and protein-level changes associated with foam cell formation under conditions of pharmacological modulation of the geranylgeranylation pathway, without directly manipulating KLF2 expression or activity.

### Quantitative real-time PCR

Total RNA was extracted from cells using the RNeasy Mini Kit (Qiagen) following the manufacturer’s instructions. The purity and concentration of RNA were determined using a NanoDrop spectrophotometer (Thermo Scientific). Complementary DNA (cDNA) was synthesized from 1 μg of total RNA using the High-Capacity cDNA Reverse Transcription Kit (#4387406, Applied Biosystems).

Quantitative real-time PCR was performed using SYBR Green Master Mix (#4309155, Applied Biosystems) on a Quant Studio 5 Real-Time PCR System (Thermo Fisher Scientific). Target genes included key autophagy markers (ATG5, ATG7, Beclin1, LC3B) and mitophagy-related genes (Drp1, Fis1, Parkin, and Pink1) (Primers were shown in Supplementary Table 1). GAPDH was used as the internal control for normalization. All reactions were run in triplicate, and relative gene expression levels were calculated using the 2^−ΔΔCt^ method. Results were expressed as fold changes relative to the control group.

### Immunocytochemistry and confocal microscopy

For immunofluorescence analysis, RAW264.7 cells were seeded on sterile glass coverslips placed in 6-well plates and treated as described in experimental conditions. Following incubation, cells were fixed with 4% paraformaldehyde for 15 minutes at room temperature to preserve cellular architecture. After fixation, cells were permeabilized using 0.1% Triton X-100 for 10 minutes and then blocked with 5% bovine serum albumin (BSA) for 1 hour to reduce non-specific binding. Cells were incubated overnight at 4 °C with primary antibodies targeting autophagy (Atg5, Atg7, Beclin1, and LC3B), mitochondrial fission (Drp1 and Fis1) and mitophagy-related proteins, including Parkin and Pink1 (Supplementary Table 2). The following day, coverslips were washed thoroughly with 1 × PBS and incubated in the dark for 45 minutes at room temperature with species-appropriate Alexa Fluor-conjugated secondary antibodies, either Alexa Fluor 488 (#A11001) or Alexa Fluor 647 (#A21235) from Invitrogen, at a dilution of 1:2000. After secondary antibody incubation, cells were washed three times with PBS and counterstained with DAPI to visualize nuclei. Coverslips were mounted onto glass slides using ProLong Gold Antifade Reagent (Thermo Fisher) and sealed. High-resolution fluorescence images were captured using a Leica Stellaris 8 Falcon STED super-resolution confocal microscope (Germany) equipped with a 100 × oil immersion objective. For quantitative analysis, five randomly selected fields per coverslip were imaged, and fluorescence intensity was quantified using ImageJ (NIH) following background subtraction. Mean fluorescence intensity was measured on a per-cell basis, normalized to the control group, and averaged across three independent biological experiments to ensure reproducibility.

### Glycolysis stress test

Glycolytic function in RAW264.7 macrophages was assessed using the Seahorse XF Glycolysis Stress Test Kit (#103020–100, Agilent Technologies) following the manufacturer’s protocol. Cells were seeded at a density of 25,000 cells per well in XF96 cell culture microplates and allowed to adhere overnight. Before the assay, cells were treated with experimental compounds as required, then washed and incubated for 1 hour at 37 °C in a non-CO_2_ incubator with Seahorse XF assay medium - glucose-free DMEM supplemented with 2 mM glutamine.

The assay measured extracellular acidification rate (ECAR) as a proxy for glycolytic flux. This was done through sequential injections of: D-glucose (10 mM) to initiate glycolysis, Oligomycin A (1 μM) to inhibit ATP synthase and force cells to rely solely on glycolysis for energy, thereby revealing glycolytic capacity, and 2-deoxy-D-glucose (2-DG, 50 mM), a glucose analog that inhibits glycolysis, to assess non-glycolytic acidification. The doses of oligomycin were chosen based on prior dose–response optimization (range tested: 0.125–2 μM), and the minimum dose that achieved a maximal ECAR response was selected for this study. ECAR values obtained from the Seahorse XF glycolysis stress test was normalized per well based on cell number (25,000 cells per well) to account for differences in cell density across conditions. The assay was designed to capture key glycolytic parameters as follows: Basal glycolysis = ECAR after glucose injection – ECAR after 2-DG. Glycolytic capacity = ECAR after oligomycin injection – ECAR after 2-DG. Glycolytic reserve = ECAR after oligomycin – ECAR after glucose. After completing the assay, the protein content in each well was quantified using a bicinchoninic acid (BCA) protein assay, and the ECAR values were normalized accordingly. All data were analyzed using the Seahorse Wave software version 2.6.1 (Agilent Technologies).

### Statistical analysis

All experiments were performed in triplicate (n = 3), and data were presented as mean ± standard deviation (SD). Statistical comparisons between groups were performed using one-way ANOVA, followed by Tukey's multiple comparison test using GraphPad Prism 9 software. A p-value of <0.05 was considered statistically significant.

## Results

### Effect of reduced KLF2 on autophagy and mitophagy-related gene expressions during FC formation

**Fig. 1.**
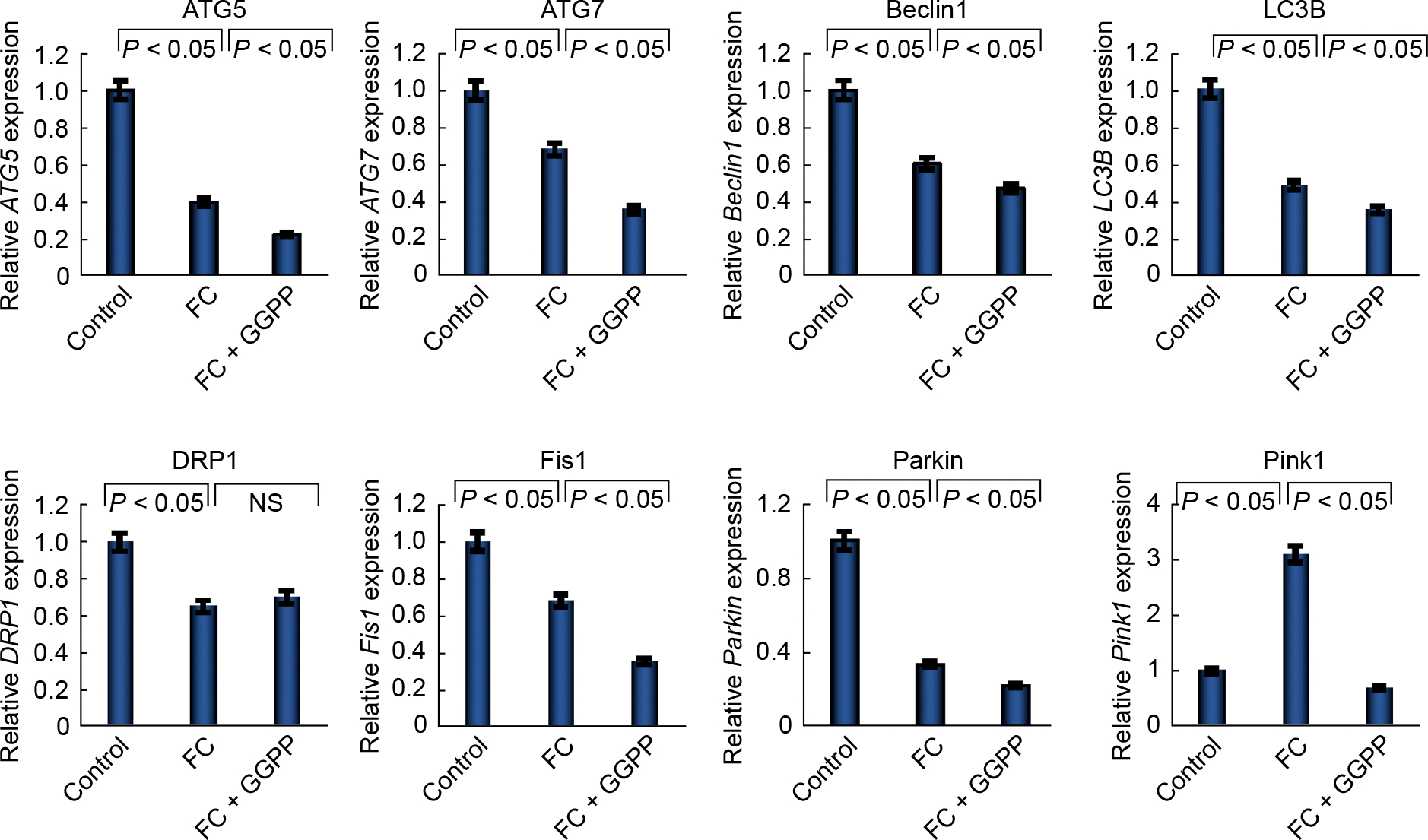
Suppression of KLF2 further decreased autophagy and mitophagy-related gene expressions. Gene expression analysis by qRT-PCR showing the fold changes of ATG5 ( ~ 0.65-fold), ATG7 ( ~ 0.52-fold), Beclin1 ( ~ 0.68-fold), LC3B ( ~ 0.74-fold), Drp1 ( ~ 0.60-fold), Fis1 ( ~ 0.57-fold), Parkin ( ~ 0.48-fold), and Pink1 ( ~ 3.5-fold) in oxLDL-treated RAW264.7 macrophages ± GGPP (KLF2 downregulation). Values are normalized to control (1.0). Data are expressed as mean ± SEM of three independent experiments (n = 3), each in triplicate. Statistical significance was assessed by one-way ANOVA with Tukey's post hoc test.

To evaluate the role of KLF2 in regulating autophagy and mitophagy during FC formation, we measured mRNA levels of autophagy-related genes such as ATG5, ATG7, Beclin1 (BECN), and microtubule-associated protein 1 light chain 3 beta (LC3B), as well as mitochondrial fission such as dynamin-related protein 1 (Drp1) and Fis1, and mitophagy-related genes (Parkin, and Pink1) in RAW264.7 cells. These proteins are essential for detecting, isolating, and removing damaged mitochondria. GGPP further reduced the expression of most autophagy and mitophagy markers, including Beclin1, LC3B, Parkin, and Pink1, whereas DRP1 expression remained unchanged (Fig. 1). Notably, co-treatment with the KLF2 inhibitor GGPP caused a further reduction in the expression levels of most of the markers mentioned above (*P* < 0.05), suggesting that KLF2 helps maintain mitochondrial function during FC formation (Fig. 1).

### Effect of induced KLF2 on autophagy and mitophagy-related gene expressions during FC formation

**Fig. 2.**
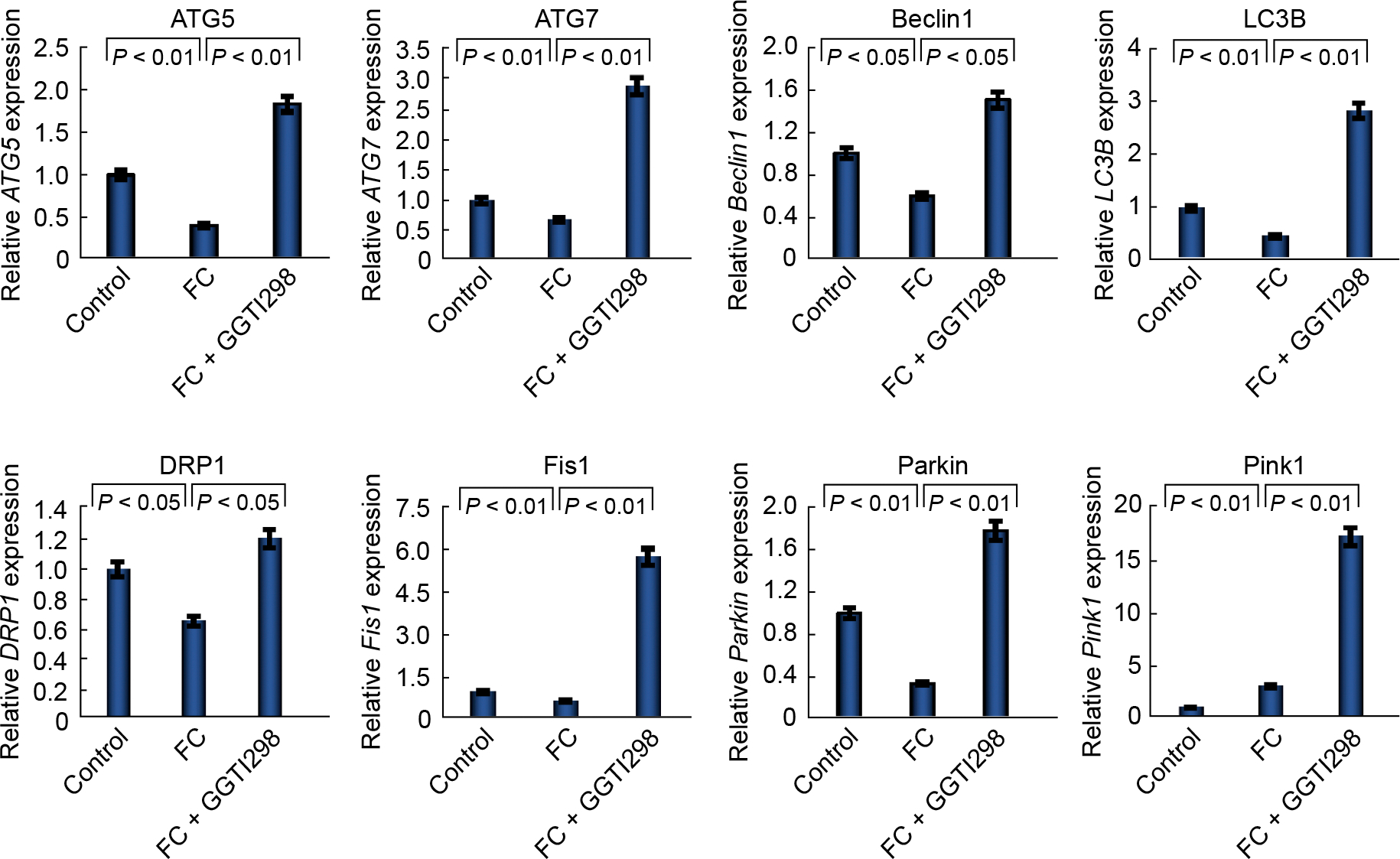
Induction of KLF2 elevated autophagy and mitophagy-related gene expressions. Quantitative RT-PCR analysis was performed to evaluate gene expression changes in RAW264.7 cells during foam cell formation induced by ox-LDL in the presence of GGTI-298. Treatment with GGTI-298 upregulated autophagy-related genes (ATG5: 2.1-fold; ATG7: 1.8-fold; Beclin1: 1.6-fold; LC3B: 2.3-fold), mitochondrial fission markers (Drp1: 1.5-fold; Fis1: 1.4-fold), and mitophagy-related genes (Parkin: 3.2-fold; Pink1: 3.9-fold), compared to ox-LDL-only treatment. Data represent mean ± SEM from three independent experiments (n = 3), with statistical analysis performed using one-way ANOVA followed by Tukey’s post hoc test (*P* < 0.05).

To explore the effect of KLF2 activation, we added GGTI298 to the myeloid cells during the formation of FCs. Results recapitulated that the cells exposed to ox-LDL alone displayed significant suppression of autophagy and mitophagy-related gene expressions. However, after the addition of GGTI298, we found that the significantly increased (*P* < 0.05) the transcript levels of ATG5, ATG7, Beclin1, LC3B, Drp1, Fis1, Parkin, and Pink1 compared to the ox-LDL-added cells (Fig. 2). These results suggest that the activation of KLF2 can reestablish disrupted mitochondrial maintenance mechanisms and support cellular responses, reinforcing its protective function in limiting foam cell-associated dysfunction.

### Effect of reduced KLF2 on autophagy-related markers during FC formation

**Fig. 3.**
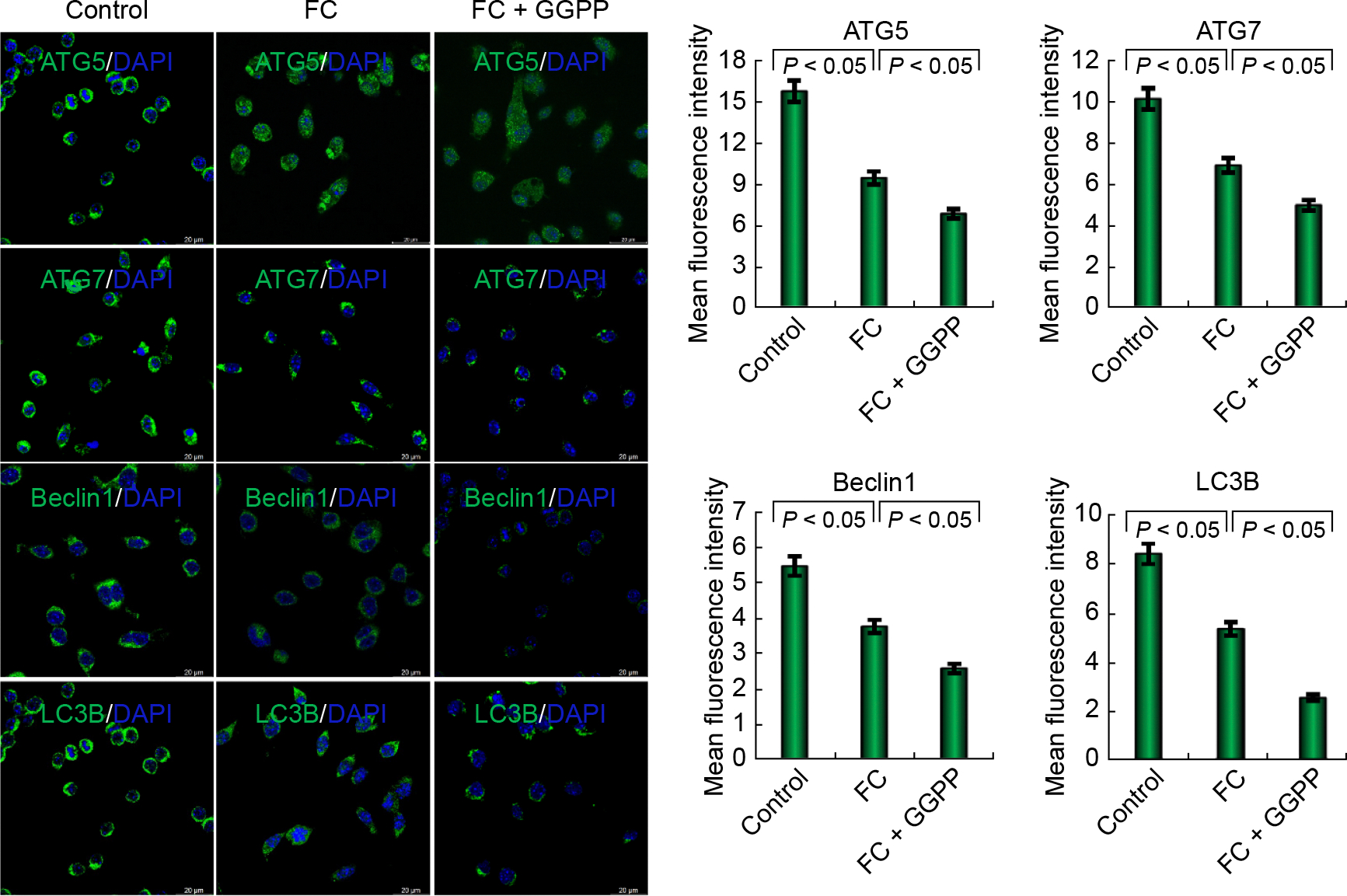
Suppression of KLF2 further decreased the fluorescence intensity of the autophagy-related molecules. Confocal microscopy images and quantitative fluorescence intensity were performed for autophagy markers, including ATG5, ATG7, Beclin1, and LC3B. Ox-LDL–induced foam cell formation is associated with reduced fluorescence intensity of ATG5, ATG7, Beclin1, and LC3B compared with control cells. GGPP treatment further decreases the relative fluorescence intensity of these autophagy-related markers under FC conditions and may indirectly influence KLF2-dependent pathways. Representative images were shown, and data were represented as mean ± SEM from three independent experiments with 1 × 10^5^ cell numbers. Statistical significance was determined using one-way ANOVA followed by Tukey’s post hoc test.

To assess the impact of KLF2 on autophagic activity during FC formation, we conducted immunocytochemical analysis to measure the levels of key autophagy markers, such as Atg5, Atg7, Be-clin1, and LC3B, in RAW264.7 cells. Cells treated with ox-LDL for FC differentiation showed a significant decrease in fluorescence intensity for all four autophagy marker proteins (*P* < 0.05) compared to untreated controls, indicating impaired autophagic flux. This impairment may lead to the buildup of damaged organelles and lipids, promoting FC development. Notably, co-treatment with GGPP intensified the reduction in autophagy maker levels (*P* < 0.05) (Fig. 3). The more pronounced downregulation with GGPP suggests that endogenous KLF2 helps protect autophagy-related proteins. This suppression of autophagy could impair the cell’s ability to clear lipid droplets and dysfunctional mitochondria, fostering lipid overload, increasing oxidative stress, and ultimately causing dysfunction in myeloid cells, which are hallmarks of early plaque formation.

### Effect of induced KLF2 on fluorescence intensity of autophagy-related molecules during FC formation

**Fig. 4.**
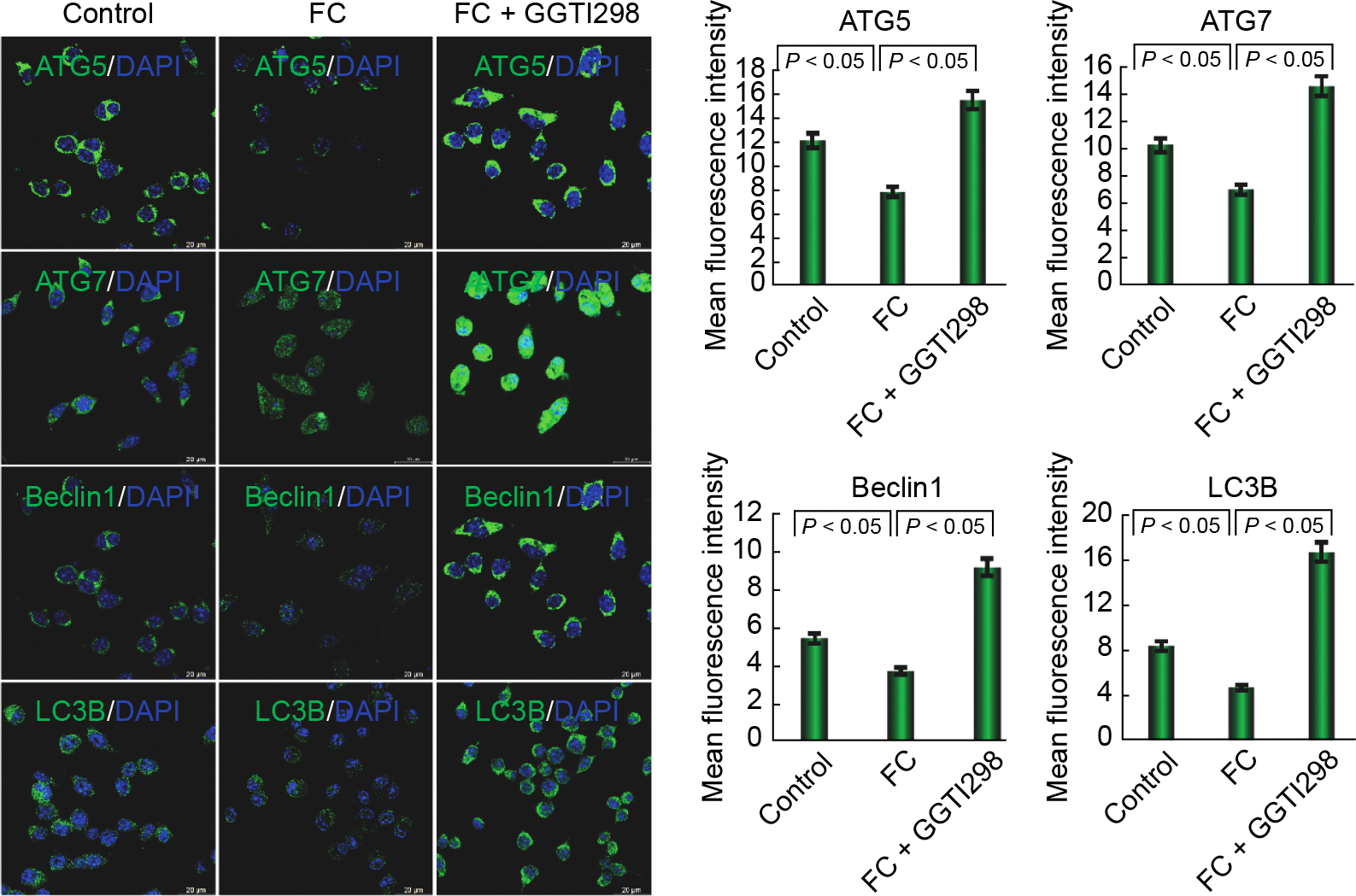
Induction of KLF2 increased fluorescence intensity of autophagy-related molecules. Confocal microscopy images and quantitative fluorescence intensity were performed for autophagy markers, including ATG5, ATG7, BECLIN1, and LC3B, and shown as images and graphically during FC formation with ox-LDL in the presence of GGTI298. Representative images were shown, and data were represented as mean ± SEM from three independent experiments with 1*10^5 cell numbers. Brackets indicate pairwise comparisons between Control vs FC and FC vs FC + GGTI298, with *P* < 0.05 considered statistically significant. Statistical significance was determined using one-way ANOVA followed by Tukey’s post hoc test.

To determine whether activation of KLF2 can counteract the suppression of autophagy during FC formation, we examined the protein levels of ATG5, ATG7, BECLIN1, and LC3Bin RAW264.7 cells after adding GGTI298 to the myeloid cells. We observed that the addition of GGTI298 caused a significant increase (*P* < 0.05) in the protein expression levels of all four autophagy markers compared to FCs alone, as shown by enhanced fluorescence signals (Fig. 4). This restoration indicates that KLF2 activation not only restores autophagic capacity but also enhances the cells’ ability to handle intracellular lipid load and remove damaged cytoplasmic components. The upregulation of LC3B, a key autophagosome formation marker, suggests a revival of autophagy, while the increase in Beclin1, an essential regulator of autophagosome initiation, highlights a coordinated reactivation of the autophagy machinery at the marker level (Fig. 4). These findings support the idea that KLF2 acts as a transcriptional regulator of autophagy-related genes, and activating KLF2 through GGTI298 may help maintain myeloid cell homeostasis.

### Effect of decreased KLF2 activity on mitophagy-related molecule expression during FC formation

**Fig. 5.**
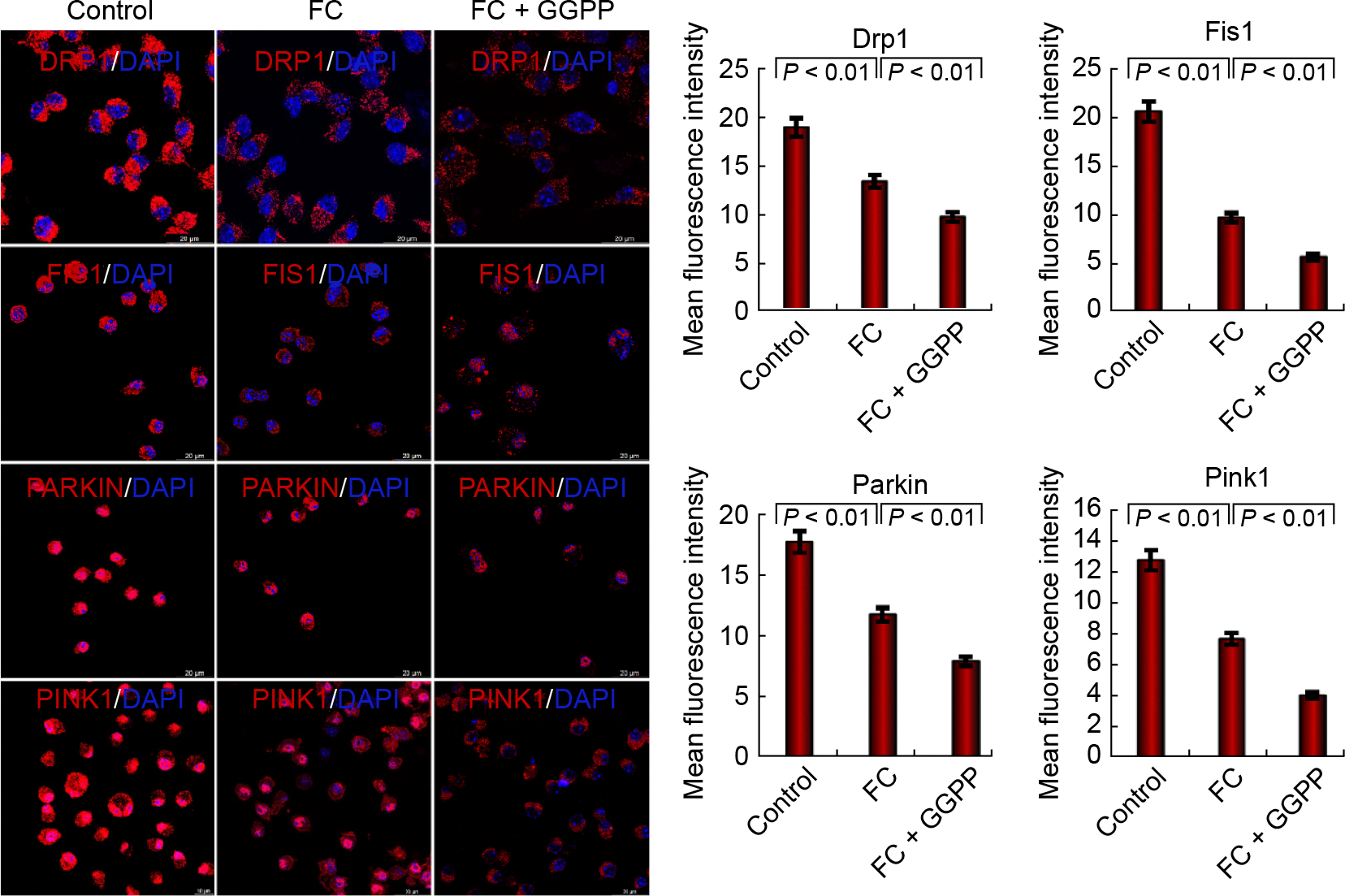
Decreased KLF2 further reduced fluorescence of mitophagy-related molecules. Confocal images were acquired from RAW264.7 macrophages using a confocal laser-scanning microscope equipped with a 100 × oil-immersion objective. Autophagy/mitophagy-related molecules were visualized in red, nuclei were counterstained with DAPI (blue), and all images were captured using identical acquisition settings across experimental groups. Scale bar = 20 μm. Confocal microscopy images were acquired, and fluorescence intensities quantified for mitochondrial fission (DRP1 and FIS1), and mitophagy markers, includingPARKIN and PINK1, and shown as images and graphically during FC formation with ox-LDL in the presence of GGPP. Representative images were shown, and data were represented as mean ± SEM from three independent experiments. The measurements were obtained from multiple randomly selected fields and cells per condition. Statistical significance was determined using one-way ANOVA followed by Tukey’s post hoc test.

We further examined the expression levels of mitophagy-related marker proteins, including DRP1, FIS1, PARKIN, and PINK1, in RAW264.7 cells under foam cell-inducing conditions. We found that the exposure to ox-LDL alone led to a substantial reduction (*P* < 0.05) in the levels of these key mitophagy regulators (Fig. 5), suggesting a potential suppression of mitophagy and mitochondrial turnover. When KLF2 was inhibited using GGPP, this decline in the expression was further exacerbated (*P* < 0.05), highlighting a deeper disruption in mitochondrial function. The diminished expression of PARKIN and PINK1points to an impaired mitochondrial damage-sensing and clearance pathway, which can result in the accumulation of dysfunctional mitochondria and increased ROS production^[28,29]^. Similarly, downregulation of DRP1 and FIS1suggests defective mitochondrial fission, a prerequisite for mitophagy^[17]^. These findings emphasize the essential role of KLF2 in sustaining mitochondrial integrity in myeloid cells by regulating gene expression and translating it to proteins that engage in the regulation of mitophagy.

### Effect of increased KLF2 on autophagy-related molecules during FC formation

**Fig. 6.**
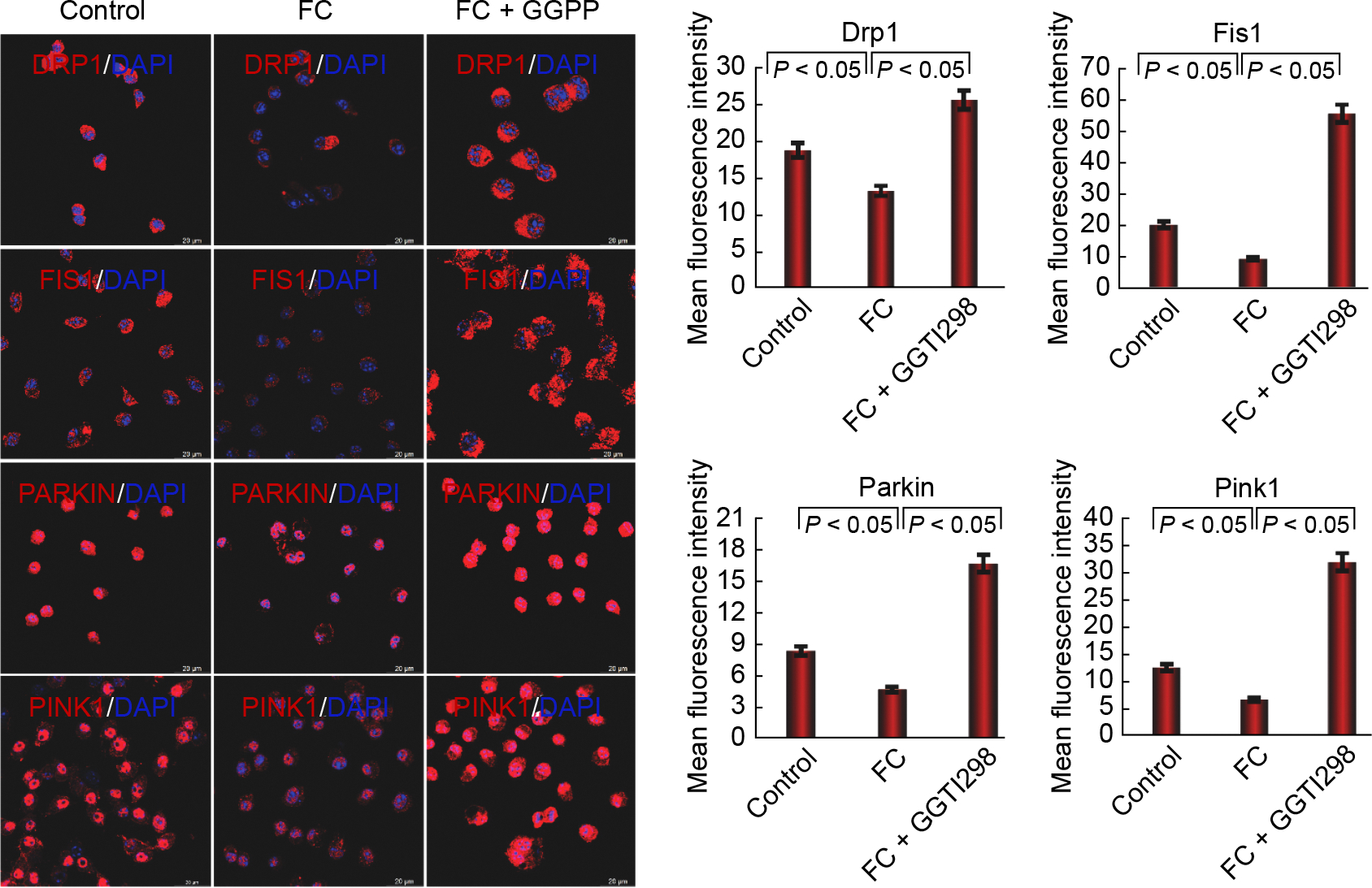
Increased KLF2 enhanced fluorescence of mitophagy-related molecules. Confocal images were acquired from RAW264.7 macrophages using a confocal laser-scanning microscope equipped with a high-magnification objective. Target proteins were visualized in red, and nuclei were counterstained with DAPI (blue). All images were captured using identical acquisition settings, including exposure and thresholding, across experimental groups. Scale bar = 20 μm. Confocal images were acquired, and fluorescence intensities quantified for mitochondrial fission (DRP1 and FIS1), and mitophagy markers, including PARKIN and PINK1, and shown as images and graphically during FC formation with oxidized low-density lipoprotein (ox-LDL) in the presence of GGTI298. Data are presented as mean ± SEM. Statistical significance was assessed using one-way ANOVA followed by Tukey’s multiple-comparisons post hoc test, with brackets indicating the specific pairwise comparisons between Control vs FC and FC vs FC + GGTI-298. A value of *P* < 0.05 was considered statistically significant.

To determine whether enhancing KLF2 activity could reverse mitophagy suppression, we added GGTI298 to the ox-LDL-challenged RAW264.7 cells, and examined the expression of DRP1, FIS1, PARKIN, and PINK1levels using confocal microscopy and evaluated the staining intensity. Immunocytochemical analysis revealed a significant restoration (*P* < 0.05) in the levels of all four proteins compared to the ox-LDL-added cells (Fig. 6). In many cases, the levels returned to those observed in untreated control macrophages, indicating that KLF2 activation reinitiates the mitophagy pathway. The re-expression of Pink1 and Parkin implies a revival of mitochondrial damage recognition and clearance mechanisms, while the increased levels of DRP1 and FIS1suggest restoration of mitochondrial fission necessary for mitophagic vesicle formation^[30]^. These changes support the hypothesis that KLF2 directly or indirectly enhances transcription and/or translation of genes critical for mitochondrial surveillance and autophagy. Altogether, this evidence positions KLF2 as a central regulator of myeloid cell resilience against lipid-induced mitochondrial dysfunction, offering therapeutic value in early-stage management of FC development, which is the causal factor for atherosclerosis development by preserving mitochondrial dynamics and preventing pro-inflammatory activation.

### Effect of decreased KLF2 on glycolytic parameters during oxLDL-induced FC formation

**Fig. 7.**
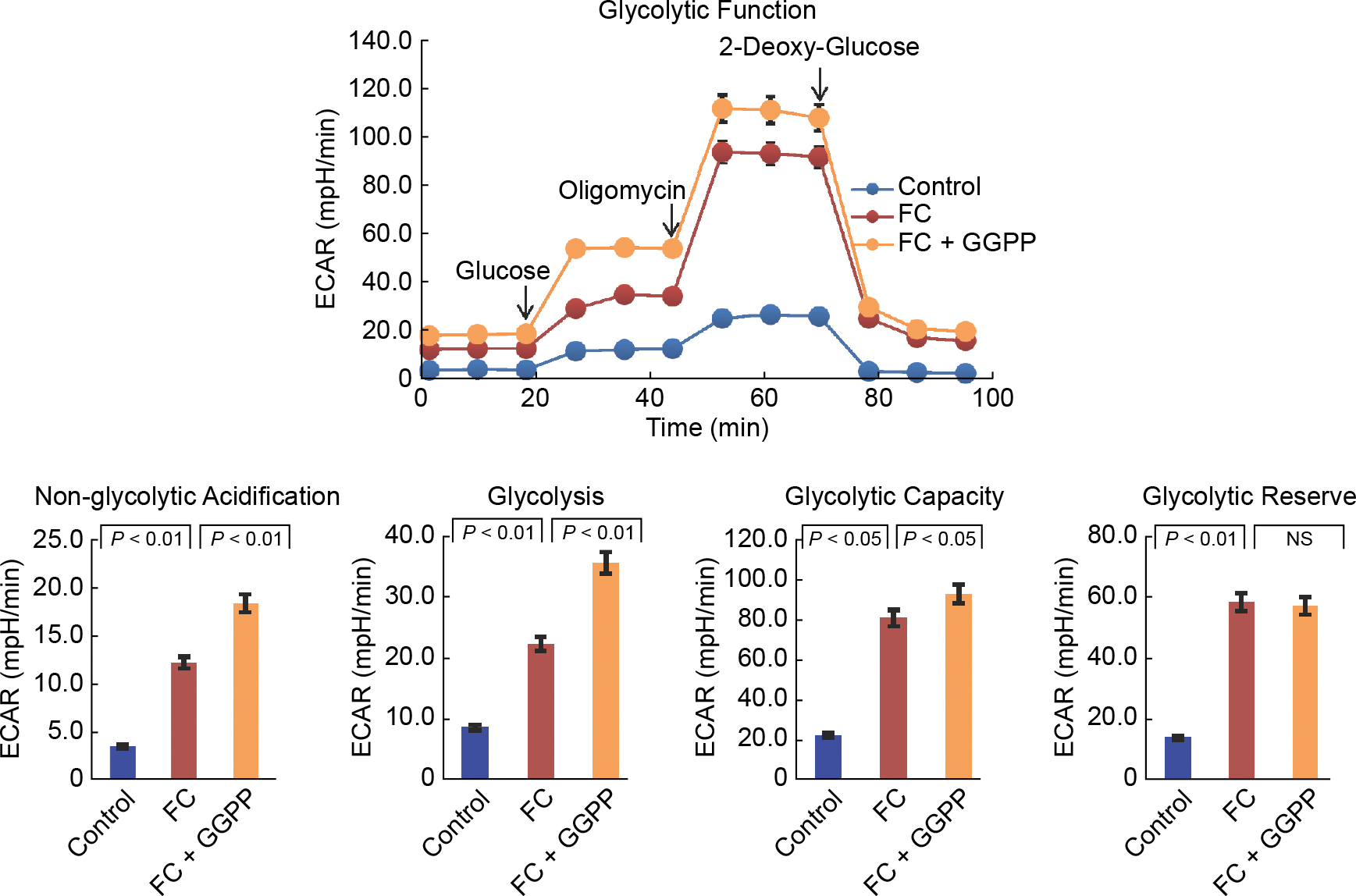
Decreased KLF2 further increased the glycolysis functions in myeloid cells during FC formation. A Seahorse XF glycolysis stress test was performed in RAW264.7 macrophages undergoing ox-LDL–induced foam cell formation with or without GGPP. Brackets on the bar graphs explicitly indicate the pairwise comparisons tested (e.g., Control vs FC; FC vs FC + GGPP), and “NS” is defined as not statistically significant. Data are presented as mean ± SEM, and statistical significance was determined using one-way ANOVA with Tukey’s multiple-comparisons test.”

To assess the role of KLF2 in glycolytic metabolism during FC development, initially, we have inhibited KLF2 levels after adding GGPP during FC formation of RAW264.7 cells, and measured the key parameters of glycolysis, such as non-glycolytic acidification, basal glycolysis, glycolytic capacity, and glycolytic reserve using flux analysis. Results show that the FC formation after the addition of oxidized LDL (ox-LDL) to the myeloid cells significantly elevated all four parameters; this metabolic reprogramming was further amplified in myeloid cells after the addition of GGPP to the culture, suggesting that the inhibition of KLF2 intensifies the glycolytic flux (Fig. 7). These results indicate that a metabolic shift toward increased glycolytic activity is associated with FC formation.

### Effect of increased KLF2 on glycolytic mitochondrial function during FC formation

**Fig. 8.**
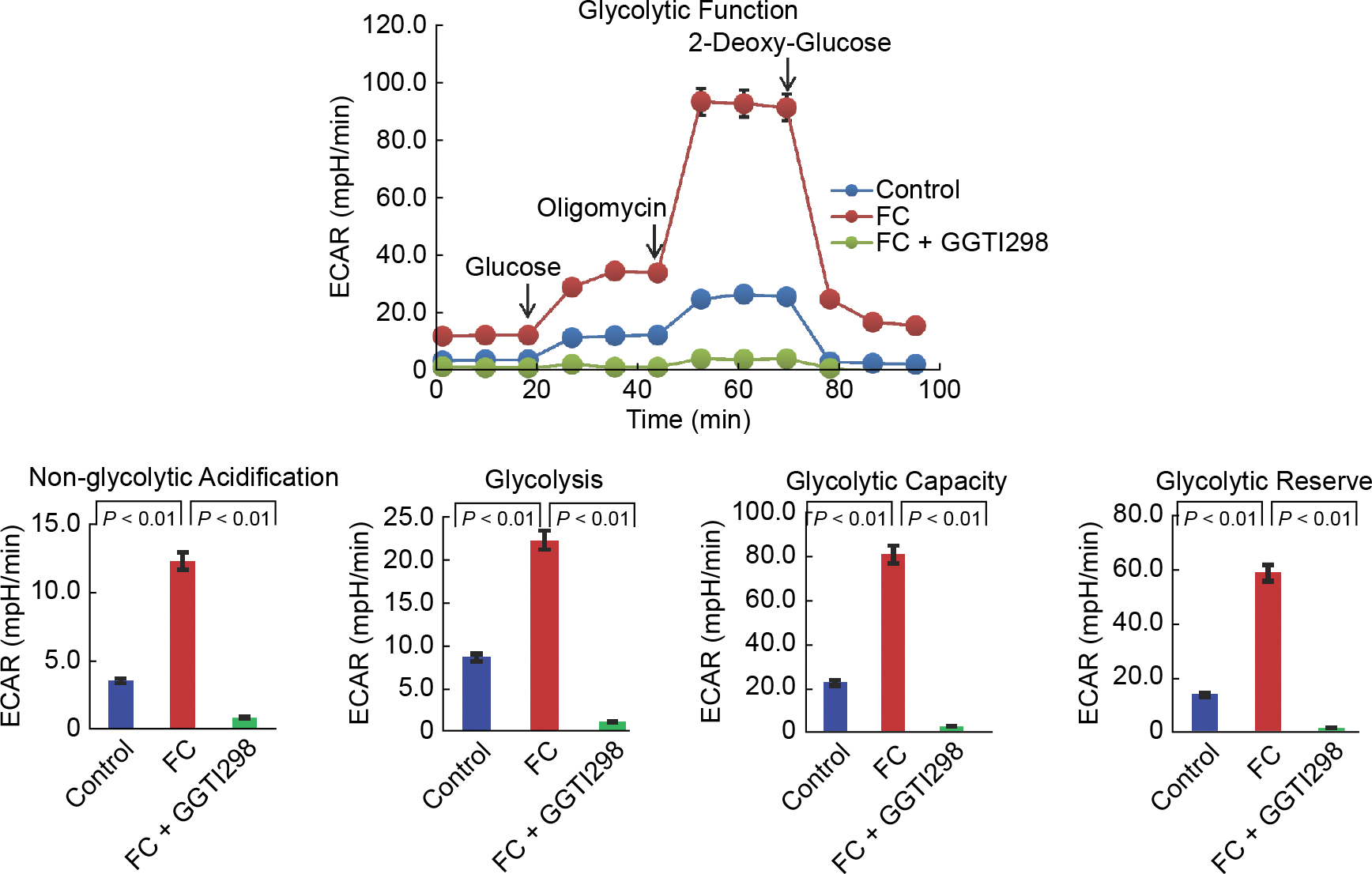
Increased KLF2 reduced the glycolysis functions in myeloid cells during FC formation. Seahorse analysis was performed in RAW264.7 cells during the FC formation with ox-LDL in the presence of GGTI298, and data were presented in graphical form. Brackets on the bar graphs now indicate the specific pairwise comparisons tested (Control vs FC; FC vs FC + GGTI298), *P* < 0.01 is defined as statistically significant, and “NS” is explicitly defined as not significant. Data represented as mean ± SEM from three independent experiments. Statistical significance was determined using one-way ANOVA followed by Tukey’s post hoc test.

To assess the role of KLF2 in glycolytic metabolism during FC development, we next activated the KLF2 levels after adding GGTI298 during FC formation of RAW264.7 cells, and measured the key parameters of glycolysis, such as non-glycolytic acidification, basal glycolysis, glycolytic capacity, and glycolytic reserve using flux analysis. In contrast, activation of KLF2 after the addition of GGTI298 to the culture markedly suppressed these glycolytic responses (Fig. 8). These exhibited reduced levels of glycolytic activity, in some cases approaching or falling below those observed in untreated control cells. These findings highlight the regulatory function of KLF2 in maintaining metabolic balance during FC formation by limiting excessive glycolysis, which is commonly associated with pro-inflammatory polarization of myeloid cells and the progression of FC formation.

## Discussion

Autophagy is a conserved catabolic process that involves the sequestration of cytoplasmic components, including damaged proteins and organelles, into autophagosomes, which subsequently fuse with lysosomes for degradation and recycling. This pathway is essential for maintaining intracellular homeostasis, particularly under stress conditions such as nutrient deprivation, oxidative stress, or lipid overload^[31]^. A specialized form of autophagy, mitophagy, specifically targets dysfunctional mitochondria for clearance, and is crucial for preventing mitochondrial ROS accumulation and preserving mitochondrial integrity^[32]^.

Importantly, both autophagy and mitophagy intersect with DNA damage response pathways. Cells encountering genotoxic stress, such as double-strand breaks or oxidative lesions, activate checkpoint kinases that not only halt cell cycle progression but also initiate repair. Autophagy facilitates this process by removing cytosolic DNA fragments and damaged organelles that could otherwise amplify inflammatory signals^[33]^. In addition, mitophagy helps limit ROS-induced oxidative DNA damage by maintaining healthy mitochondria. Proteins such as PINK1 and Parkin are critical to this process; PINK1 accumulates on the outer membrane of depolarized mitochondria, recruiting Parkin, which ubiquitinates mitochondrial proteins to target them for autophagic degradation^[34]^. Therefore, both autophagy and mitophagy serve as frontline mechanisms in preventing genotoxic stress and promoting genomic stability.

Moreover, our study sheds light on the underappreciated role of KLF2 in regulating autophagy and mitophagy. It was previously shown that KLF2 plays a critical role in osteoclastic differentiation of myeloid cells^[23,24]^. However, it was not known whether KLF2 is involved in regulating autophagy and mitophagy during FC formation of myeloid cells. Herein, we showed that ox-LDL significantly downregulated the expression of key autophagy-related genes such as ATG5, Beclin1, and LC3B, as well as mitophagy markers like Pink1 and Parkin. Our findings are in agreement with the previous report, showing that impaired autophagic flux contributes to lipid accumulation and inflammation in FCs^[6]^. We further showed that the direct involvement of KLF2 studied both loss-of-function and gain-of-function approaches. Our study showed that the inhibition of KLF2 exacerbated these inhibitory effects of autophagic and mitophagic molecules, while activation of KLF2 restored autophagy and mitophagy markers to near-control levels. These results are in parallel to previous findings, which demonstrated that KLF2 activation enhances autophagic responses in vascular smooth muscle cells^[15]^. At the molecular level, our immunocytochemical analyses confirmed and established these trends, showing that KLF2 activation re-established the expression of both early and late autophagy marker molecules that can be functional for repairing the stress-mediated cellular damage. Restoration of Pink1 and Parkin suggests that KLF2 also might play a critical role in preventing mitochondrial dysfunction and excessive ROS production by facilitating mitophagy. Previous study emphasized the importance of Pink1 stabilization in marking depolarized mitochondria for degradation, a process that, if disrupted, contributes to cellular oxidative stress and damage^[34]^.

In addition to autophagy and mitophagy, we have studied the mitochondrial function, as it is known that FC formation from monocytes upon prolonged exposure to ox-LDL undergoes a metabolic shift from oxidative phosphorylation to aerobic glycolysis, a phenomenon reminiscent of the “Warburg effect” typically seen in activated immune cells^[35]^. This shift requires rapid generation of ATP and expression of pro-inflammatory genes but compromises mitochondrial function, disrupts redox homeostasis, and hampers cellular repair mechanisms. We observed that oxLDL exposure markedly increased glycolytic flux, including elevated basal glycolysis and glycolytic reserve. Interestingly, when KLF2 expression was inhibited, this cellular glycolytic function was exacerbated, whereas activation of KLF2 significantly reversed these effects. These observations are in agreement with previous studies, where it was demonstrated that transcriptional control plays a critical role in metabolic reprogramming of inflammatory myeloid cells^[36]^. Our data suggest that KLF2 acts as a negative transcriptional regulator of pro-atherogenic glycolysis, promoting an active quiescent metabolic profile in myeloid cells under the condition of elevated oxidative stress.

The interplay between autophagy/mitophagy and glycolysis is increasingly recognized as a fundamental component of cellular adaptation to stress. Excessive glycolysis, driven by inflammatory stimuli, can lead to mitochondrial dysfunction and elevated ROS production^[6]^. These ROS, in turn, can damage mitochondrial DNA, lipids, and proteins, perpetuating a cycle of oxidative stress. Under normal circumstances, mitophagy would clear such damaged mitochondria; however, glycolysis-induced inhibition of autophagy-related gene expression may impair this feedback control, contributing to mitochondrial and cellular dysfunction^[7]^. Our findings show that KLF2 serves as a metabolic checkpoint that suppresses glycolysis while simultaneously promoting autophagy and mitophagy, thus maintaining normal mitochondrial function and preventing ROS-induced damage.

Furthermore, emerging evidence indicates a bidirectional relationship between glycolysis and autophagy that plays a critical role in FC formation. Notably, enhanced glycolytic activity has been shown to suppress autophagy, thereby contributing to FC formation. It was shown that the inhibition of glycolysis using 3PO reduces intracellular levels of fructose 2,6-bisphosphate and attenuates coronary artery plaque formation in mice, while also improving cardiac function by downregulating the NF-κB/TNF-α signaling pathway and decreasing autophagy/mitophagy^[37]^, which supports our hypothesis. These findings support the notion that aberrant glucose metabolism can directly inhibit autophagic processes, thereby exacerbating vascular inflammation and plaque development. KLF2 thus serves as a crucial integrator of autophagic pathways and metabolic activities in myeloid cell lines, shaping their mitochondrial integrity and function during FC formation.

Our findings highlight that KLF2 directly influences the autophagic machinery by modulating the expression of Beclin1 and LC3B. Beclin1 is a central initiator of autophagosome nucleation through the PI3K class III complex, while LC3B is required for membrane elongation and autophagosome maturation. In the presence of ox-LDL, both *Beclin1* and *LC3B* were significantly suppressed, consistent with impaired autophagic flux observed during foam cell formation. Notably, KLF2 inhibition with GGPP further exacerbated this suppression, while KLF2 activation with GGTI-298 restored Beclin1 and LC3B expression to near-control levels. This axis is consistent with previous reports showing that KLF2 transcriptionally upregulates Beclin1 and LC3B in vascular and stem cell contexts, thereby maintaining cellular homeostasis under metabolic stress^[23,25]^. Our data extend these observations into macrophage foam cells, providing evidence that KLF2 regulates the Beclin1-LC3B pathway as part of its protective role in maintaining autophagic flux and mitochondrial quality control during early atherosclerosis. Together, these results position KLF2 as a central transcriptional checkpoint integrating inflammatory signals, autophagy regulation, and mitochondrial health.

These results expand upon earlier demonstrations of KLF2 as an anti-inflammatory and vasoprotective regulator. For instance, it was shown that KLF2 is a critical flow-responsive gene that confers endothelial protection by regulating thrombomodulin and eNOS expression^[38]^. Our data now suggest that KLF2 exerts a similar regulatory function in myeloid cells during FC formation by integrating autophagic, mitochondrial signals, and metabolic functions, which is in agreement with the previous report^[38]^.

## Conclusion

In conclusion, this study positions KLF2 as a master regulator that coordinates anti-inflammatory metabolic programming, autophagic flux, and controls mitochondrial function during FC formation. Given its multifaceted role, therapeutic strategies aimed at enhancing KLF2 activity, either directly through small molecules, such as GGTI298, or indirectly via epigenetic modulation, could offer promising avenues for early intervention during FC formation, which is a critical early event for the pathogenesis of atherosclerosis.

## Supplementary Material

Supplementary Materials

## Abbreviations

2-DG: 2-deoxy-D-glucose
ANOVA: analysis of variance
ATG: autophagy-related gene
ATP: Adenosine triphosphate
BCA: bicinchoninic acid
BECN: Beclin1
BSA: bovine serum albumin
cDNA: complementary deoxyribonucleic acid
DMEM: dulbecco’ s modified eagle medium
Drp1: dynamin-related protein 1
ECAR: extracellular acidification rate
eNOS: endothelial nitric oxide synthase
FC: foam cells
FIS1: fission mitochondrial 1
GGPP: geranylgeranyl pyrophosphate
GGTI298: geranylgeranyltransferase I (GGTase I) inhibitor 298
KLF2: Krüppel-like factor 2
LC3B: microtubule-associated protein 1 light chain 3 beta
NF-κB,: nuclear factor kappa-light-chain-enhancer of activated B cells
ox-LDL: oxidized low-density lipoprotein
PI3K: phosphatidylinositol 3-kinases
PINK1: PTEN-induced kinase 1
real-time PCR: real-time polymerase chain reaction
ROS: reactive oxygen species
TNF-α: tumor necrosis factor-α.

## References

[1] Libby P, Ridker PM, Hansson GK. Progress and challenges in translating the biology of atherosclerosis. Nature, 2011, 473(7347): 317–325. 21593864 10.1038/nature10146

[2] Moore KJ, Sheedy FJ, Fisher EA. Macrophages in atherosclerosis: a dynamic balance. Nat Rev Immunol, 2013, 13(10): 709–721. 23995626 10.1038/nri3520 PMC4357520

[3] Levine B, Kroemer G. Autophagy in the pathogenesis of disease. Cell, 2008, 132(1): 27–42. 18191218 10.1016/j.cell.2007.12.018 PMC2696814

[4] Ashrafi G, Schwarz T. The pathways of mitophagy for quality control and clearance of mitochondria. Cell Death Differ, 2013, 20(1): 31–42. 22743996 10.1038/cdd.2012.81 PMC3524633

[5] Palikaras K, Lionaki E, Tavernarakis N. Mechanisms of mitophagy in cellular homeostasis, physiology and pathology. Nat Cell Biol, 2018, 20(9): 1013–1022. 30154567 10.1038/s41556-018-0176-2

[6] Ouimet M, Franklin V, Mak E, Autophagy regulates cholesterol efflux from macrophage foam cells via lysosomal acid lipase. Cell Metab, 2011, 13(6): 655–667. 21641547 10.1016/j.cmet.2011.03.023 PMC3257518

[7] Sergin I, Evans TD, Zhang X, Exploiting macrophage autophagy-lysosomal biogenesis as a therapy for atherosclerosis. Nat Commun, 2017, 8(1): 15750. 28589926 10.1038/ncomms15750 PMC5467270

[8] Tall AR, Yvan-Charvet L. Cholesterol, inflammation and innate immunity. Nat Rev Immunol, 2015, 15(2): 104–116. 25614320 10.1038/nri3793 PMC4669071

[9] Liao X, Sluimer JC, Wang Y, Macrophage autophagy plays a protective role in advanced atherosclerosis. Cell Metab, 2012, 15(4): 545–553. 22445600 10.1016/j.cmet.2012.01.022 PMC3322248

[10] Li W, McIntyre TM, Silverstein RL. Ferric chloride-induced murine carotid arterial injury: A model of redox pathology. Redox Biol, 2013, 1(1): 50–55. 25101237 10.1016/j.redox.2012.11.001 PMC4116643

[11] O'Neill LA, Kishton RJ, Rathmell J. A guide to immunometabolism for immunologists. Nat Rev Immunol, 2016, 16(9): 553–565. 27396447 10.1038/nri.2016.70 PMC5001910

[12] Narendra DP, Jin SM, Tanaka A, PINK1 is selectively stabilized on impaired mitochondria to activate Parkin. PLoS Biol, 2010, 8(1): e1000298. 20126261 10.1371/journal.pbio.1000298 PMC2811155

[13] SenBanerjee S, Lin Z, Atkins GB, KLF2 Is a novel transcriptional regulator of endothelial proinflammatory activation. J Exp Med, 2004, 199(10): 1305–1315. 15136591 10.1084/jem.20031132 PMC2211816

[14] Das H, Kumar A, Lin Z, Kruppel-like factor 2 (KLF2) regulates proinflammatory activation of monocytes. Proc Natl Acad Sci USA, 2006, 103(17): 6653–6658. 16617118 10.1073/pnas.0508235103 PMC1458936

[15] Lin Z, Kumar A, SenBanerjee S, Kruppel-like factor 2 (KLF2) regulates endothelial thrombotic function. Circ Res, 2005, 96(5): e48–e57. 15718498 10.1161/01.RES.0000159707.05637.a1

[16] Atkins GB, Jain MK. Role of Kruppel-like transcription factors in endothelial biology. Circ Res, 2007, 100(12): 1686–1695. 17585076 10.1161/01.RES.0000267856.00713.0a

[17] Xu S, Wang Z, Guo F, Mitophagy in ischemic heart disease: molecular mechanisms and clinical management. Cell Death Dis, 2024, 15(12): 934. 39737905 10.1038/s41419-024-07303-3 PMC11685431

[18] Sen-Banerjee S, Mir S, Lin Z, Kruppel-like factor 2 as a novel mediator of statin effects in endothelial cells. Circulation, 2005, 112(5): 720–726. 16043642 10.1161/CIRCULATIONAHA.104.525774

[19] Naidu P, Das M, Hansda S, Mechanisms of Ellagic Acid (EA)-Mediated Osteogenic Differentiation of Human Dental Pulp-Derived Stem Cells. ACS Omega, 2025, 10(15): 15229–15242. 40290905 10.1021/acsomega.4c10642 PMC12019503

[20] Howlader MSI, Prateeksha P, Hansda S, Secretory products of DPSC mitigate inflammatory effects in microglial cells by targeting MAPK pathway. Biomed Pharmacother, 2024, 170: 115971. 38039760 10.1016/j.biopha.2023.115971

[21] Prateeksha P, Howlader MSI, Hansda S, Secretome of dental pulp-derived stem cells reduces inflammation and proliferation of glioblastoma cells by deactivating Mapk-Akt pathway. Dis Res, 2023, 3(2): 74. 38213319 10.54457/DR.202302006 PMC10783424

[22] Greene CJ, Anderson S, Barthels D, DPSC Products Accelerate Wound Healing in Diabetic Mice through Induction of SMAD Molecules. Cells, 2022, 11(15): 2352. 35954256 10.3390/cells11152409 PMC9368341

[23] Laha D, Deb M, Das H. KLF2 (kruppel-like factor 2 [lung]) regulates osteoclastogenesis by modulating autophagy. Autophagy, 2019, 15(12): 2063–2075. 30894058 10.1080/15548627.2019.1596491 PMC6844519

[24] Laha D, Sarkar J, Maity J, Polyphenolic Compounds Inhibit Osteoclast Differentiation While Reducing Autophagy through Limiting ROS and the Mitochondrial Membrane Potential. Biomolecules, 2022, 12(9): 1307. 36139058 10.3390/biom12091220 PMC9496366

[25] Maity J, Deb M, Greene C, KLF2 regulates dental pulpderived stem cell differentiation through the induction of mitophagy and altering mitochondrial metabolism. Redox Biol, 2020, 36: 101622. 32777717 10.1016/j.redox.2020.101622 PMC7417940

[26] Parmar KM, Nambudiri V, Dai G, Statins exert endothelial atheroprotective effects via the KLF2 transcription factor. J Biol Chem, 2005, 280(29): 26714–26719. 15878865 10.1074/jbc.C500144200

[27] Zhao JY, Natarajan SK, Chronos N, Cerivastatin Represses Atherogenic Gene Expression through the Induction of Klf2 Via Isoprenoid Metabolic Pathways. Cell Mol Biol Lett, 2015, 20(5): 825–839. 26556845 10.1515/cmble-2015-0049

[28] Ma C, Wu H, Yang G, Calycosin ameliorates atherosclerosis by enhancing autophagy via regulating the interaction between KLF2 and MLKL in apolipoprotein E gene - deleted mice. Br J Pharmacol, 2022, 179(2): 252–269. 34713437 10.1111/bph.15720

[29] Wu Y, Jiang T, Hua J, PINK1/Parkin-mediated mitophagy in cardiovascular disease: From pathogenesis to novel therapy. Int J Cardiol, 2022, 361: 61–69. 35594994 10.1016/j.ijcard.2022.05.025

[30] Mai S, Klinkenberg M, Auburger G, Decreased expression of Drp1 and Fis1 mediates mitochondrial elongation in senescent cells and enhances resistance to oxidative stress through PINK1. J Cell Sci, 2010, 123(6): 917–926. 20179104 10.1242/jcs.059246

[31] Levine B, Sinha SC, Kroemer G. Bcl-2 family members: Dual regulators of apoptosis and autophagy. Autophagy, 2008, 4(5): 600–606. 18497563 10.4161/auto.6260 PMC2749577

[32] Youle RJ, Narendra DP. Mechanisms of mitophagy. Nat Rev Mol Cell Biol, 2011, 12(1): 9–14. 21179058 10.1038/nrm3028 PMC4780047

[33] Xu Y, Shao B, Zhang Y. The significance of targeting lysosomes in cancer immunotherapy. Front Immunol, 2024, 15: 1308070. 38370407 10.3389/fimmu.2024.1308070 PMC10869645

[34] Narendra D, Tanaka A, Suen DF, Parkin is recruited selectively to impaired mitochondria and promotes their autophagy. J Cell Biol, 2008, 183(5): 795–803. 19029340 10.1083/jcb.200809125 PMC2592826

[35] Tannahill GM, Curtis AM, Adamik J, Succinate is an inflammatory signal that induces IL-1β through HIF-1α. Nature, 2013, 496(7444): 238–242. 23535595 10.1038/nature11986 PMC4031686

[36] Galván-Peña S, O’Neill LA. Metabolic reprograming in macrophage polarization. Front Immunol, 2014, 5: 420. 25228902 10.3389/fimmu.2014.00420 PMC4151090

[37] Perrotta P, Van der Veken B, Van Der Veken P, Partial inhibition of glycolysis reduces atherogenesis independent of intraplaque neovascularization in mice. Arterioscler Thromb Vasc Biol, 2020, 40(5): 1168–1181. 32188275 10.1161/ATVBAHA.119.313692 PMC7176341

[38] Parmar KM, Larman HB, Dai G, Integration of flow-dependent endothelial phenotypes by Kruppel-like factor 2. J Clin Invest, 2006, 116(1): 49–58. 16341264 10.1172/JCI24787 PMC1307560

