# Supplementary material for: Krüppel-like Factor 2 (KLF2) Regulates Autophagy, Mitophagy, Mitochondrial Health, and Function During Foam Cell Formation
Source: Dis Res. Author manuscript; Available in PMC 2026 Jul 3. (PMC13327729; doi:10.54457/dr.202601003)
Supplement: Supplementary Materials [file NIHMS2184906-supplement-Supplementary_Materials.pdf]

Supplementary Data

Krüppel-like factor 2 (KLF2) regulates autophagy, mitophagy, mitochondrial health, and functions during foam cell formation

Md Sariful Islam Howlader[1], Manjusri Das[1], Surajit Hansda[1], Prathyusha Naidu[1], and Hiranmoy Das[1*]

[1]Department of Pharmaceutical Sciences, Jerry H. Hodge School of Pharmacy, Texas Tech University Health Sciences Center, Amarillo, Texas, USA.

Table S1: Primers used in this study

Gene	Accession Number (NCBI)	Forward Primer (5′ → 3′)	Reverse Primer (5′ → 3′)
ATG5	NM_053069.3	CCT GAA GAT GAT GGC ACA GA	TGT TCA GTG TTG GCT TTG CT
ATG7	NM_028835.5	GCC TGA TGA GGA GTC ATC CA	TTG TTT CCA GCA GAG TCT GG
Beclin1	NM_019584.3	TGA AGC CTT TGG GAC ACT TC	CCA CTG AGT GAA CAG CAG GA
LC3B	NM_026160.4	CTC TTC CTG TAG GAG GCA GC	TCT TCA GAG AGC CAG GAG AG
Drp1	NM_001278528.2	TGA GGA GAG AGG ACT TCG GG	GTG TGT CCT GAA GAG CAG GA
Fis1	NM_001159764.1	CGG GTT AGC GTC TTC TTT GC	TCA TGA GGC GGT GTC ACT TT
Pink1	NM_001135167.2	CAG AGT CCC TCT TGC TCC TA	TGT GCT GTG GTT CAG TGA GT
Parkin	NM_001024231.1	GAT AAG AGC CTG GCA GAG GT	GAG TCC TGG ACA GGA TCT GA
GAPDH	NM_008084.3	AGA GGG AGG CTT GGA CTT GAA	GGA GTT GTC ATG GAT GAC CTT G

Table S2: Antibodies used in this study

No.	Antibody and Catalogue No.	Company	Dilutions for Immunocytochemistry
1.	ATG5 #ab12994	Abcam	1:250
2.	ATG7 #8558S	Cell signaling	1:250
3.	Beclin1 #3738S	Cell signaling	1:250
4.	LC3B #12741	Cell signaling	1:300
5.	DRP1 (#ab184247)	Abcam	1:250
6.	PINK1 (#6946S)	Cell signaling	1:200
7.	PARKIN (#2132S)	Cell signaling	1:200
8.	FIS1 (#10956-1-AP)	Proteintech	1:100
